# Impact of Different Energy Levels of Virtual Monoenergetic Reconstructions on Radiomic Features Stability in Organic Phantom Imaging Using Photon-Counting CT

**DOI:** 10.3390/tomography12070102

**Published:** 2026-07-06

**Authors:** Farroch Vahidi Noghani, Lukas T. Rotkopf, Stefan O. Schoenberg, Matthias F. Froelich, Isabelle Ayx, Alexander Hertel

**Affiliations:** 1 Department of Radiology and Nuclear Medicine, University Medical Center Mannheim, Heidelberg University, Theodor-Kutzer-Ufer 1-3, 68167 Mannheim, Germany; 2 Department of Radiology, German Cancer Research Center, Im Neuenheimer Feld 280, 69120 Heidelberg, Germany

**Keywords:** photon-counting CT, virtual monoenergetic reconstruction, radiomics, agreement, reliability, repeatability, reproducibility, concordance correlation coefficient, intraclass correlation coefficient

## Abstract

**Objectives**: This study investigates the repeatability and reproducibility of radiomic features extracted from different energy levels of virtual monoenergetic reconstruction (VMER) and polyenergetic reconstruction (PER) obtained with photon-counting computed tomography (PCCT). **Methods**: Sixteen organic phantoms were scanned twice in a test–retest format using a 120 kV tube potential and tube currents of 10, 50, and 100 mAs. After rotating the phantoms 90° around their z-axis, additional test–retest scans were performed. A PER and 16 VMERs were generated. Segmentation and extraction of 105 original radiomic features followed. The repeatability and reproducibility of these features were assessed using the concordance correlation coefficient (CCC) for agreement and the intraclass correlation coefficient (ICC) for reliability, excluding 14 shape-based features from the analysis. **Results**: On average, 85 out of 91 radiomic features from VMER showed high repeatability. Approximately 30% of features demonstrated high intra-scan and inter-scan reproducibility when comparing PER and VMER. For different energy levels of VMER, around 78% showed high intra-scan reproducibility, and 74% showed high inter-scan reproducibility. Comparing the average values of test and retest scans in both the initial and rotated states revealed that 65% of features showed high agreement and 73% high reliability for PER, while for VMER, these values were 51% and 55%, respectively. **Conclusions**: Radiomic features from VMERs showed high test–retest repeatability, whereas reproducibility across reconstruction types and widely separated energy levels was more limited. These findings suggest that energy levels should be carefully standardized when radiomic features are extracted from PCCT-derived VMER images.

## Simple Summary

This study examined how consistently radiomic features can be measured in photon-counting CT images reconstructed at different virtual monoenergetic energy levels and conventional polyenergetic images. The statistical analysis showed that these radiomic features can be stable under identical conditions, but their interchangeability becomes limited when the energy level or reconstruction type of images changes.

## 1. Introduction

Photon-counting computed tomography (PCCT) transformed radiological imaging by directly measuring individual photons, enhancing image quality. This method reduces image noise by excluding low-energy photons, resulting in higher spatial resolution, better signal-to-noise ratios, and fewer beam-hardening artifacts compared to traditional energy-integrating CT [1,2]. Early clinical studies have demonstrated the practicality and reliability of PCCT in routine diagnostics [3]. Furthermore, a defining feature of PCCT is its ability to perform spectral imaging, leveraging the energy-discrimination capabilities of its detectors. This enables reconstructing virtual monoenergetic images (VMIs) at different energy levels, providing enhanced tissue characterization and improved visualization of contrast agents. By analyzing material-specific attenuation properties, spectral imaging can differentiate between various substances, such as calcium and iodine, with greater precision. This unique capability not only improves diagnostic accuracy but also opens new avenues for advanced quantitative imaging techniques, further setting PCCT apart from conventional CT systems [1,2].

In the context of big data, the role of medical imaging has expanded far beyond the visual assessment traditionally performed by radiologists. The emergence of radiomics as a quantitative imaging tool has opened new frontiers in diagnostics [4]. By extracting and analyzing a vast array of measurable features from radiological data, radiomics offers insights beyond what is perceptible to the human eye, enhancing diagnostic precision and fostering a deeper understanding of disease characteristics [4]. The potential applications of radiomics span a wide range of medical fields, from oncology to cardiovascular and neuroimaging. In oncology, radiomics can aid in tumor characterization, prognostication, and treatment response monitoring [5,6,7], while in cardiology, it can provide insights into myocardial tissue properties and vascular pathologies [8,9,10]. Neurological applications include the identification of subtle patterns in neurodegenerative diseases or stroke imaging [11]. Despite progress, clinical integration of radiomics faces challenges, particularly instability due to variations in imaging protocols, reconstruction parameters, and segmentation methods, which can affect reproducibility and reliability [12,13,14]. When shifting from conventional energy-integrating computed tomography (EICT) to PCCT, the physical architecture of spectral data collection alters the mathematical baseline of radiomic features. While EICT-derived VMIs are hindered at low-keV levels by combining electronic noise and beam-hardening artifacts, PCCT removes electronic noise entirely. However, PCCT introduces a unique profile of high spatial resolution photon noise fluctuations. Consequently, choosing lower-energy thresholds (<50 keV) disrupts radiomic feature re-producibility across both platforms, though for fundamentally different physical reasons. This underscores the need for platform-specific keV optimization to ensure generalizable machine learning workflows [15,16].

The organic phantom dataset analyzed in the present work was acquired previously by Hertel et al. [17]. In that initial study, the test–retest, repositioning, and tube-current stability of radiomic features was evaluated exclusively for conventional polyenergetic reconstructions, demonstrating that a large proportion of features are highly stable on PCCT. However, the spectral capability of PCCT—and, in particular, the influence of the reconstructed energy level on radiomic feature stability—was not addressed. The present study therefore constitutes a secondary, extended analysis of this existing PCCT organic phantom dataset: no additional scans were performed; instead, the original raw acquisitions were used to generate polyenergetic and virtual monoenergetic reconstructions across energy levels ranging from 40 to 190 keV. Accordingly, only the polyenergetic results partially overlap with the previous publication, whereas the analyses of virtual monoenergetic reconstructions and of the impact of energy level represent the novel contribution of this work.

The aim of this study is to investigate the impact of different energy levels of virtual monoenergetic reconstruction (VMER) on the repeatability and reproducibility of radiomic features using phantom scans acquired with PCCT.

## 2. Materials and Methods

### 2.1. Study Design

In this study, 16 organic phantoms (four apples, four onions, four limes, and four kiwis) were scanned to assess the repeatability, reproducibility, agreement, and reliability of radiomic features extracted from polyenergetic reconstructions (PERs) and virtual monoenergetic reconstruction (VMER) images. All scans were performed using the first-generation whole-body dual-source PCCT system (NAEOTOM Alpha; Siemens Healthineers AG, Forchheim, Germany). This study is based on the previously acquired data from Hertel et al. [17], in which the phantom scans were originally conducted. No additional scans were performed; instead, this analysis utilized the image reconstructions obtained in the prior study.

### 2.2. CT Scans

**Figure 1:**
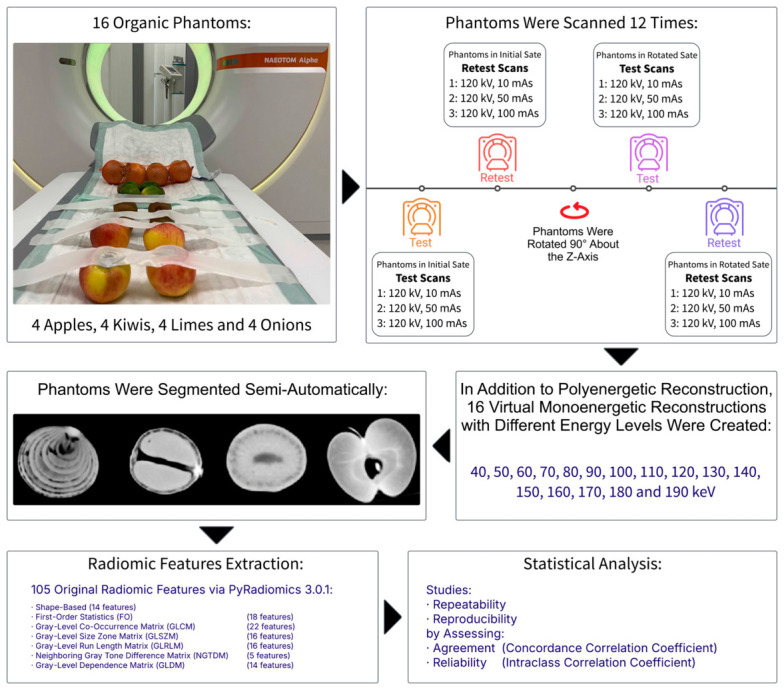
Workflow diagram of the study.

Each phantom was scanned under four different scenarios using three tube current settings: 10, 50, and 100 mAs. For all scans, the tube voltage was kept constant at 120 kV, the gantry rotation time was fixed at 0.25 s, and the nominal in-plane spatial resolution of the detector system was 0.11 mm, while images were reconstructed at 1 mm slice thickness and increment. In the first scenario, designated as the test scan, each phantom was scanned once at each of the three tube current settings. The same procedure was then repeated in the second scenario, designated as the retest scan. After completing the retest scans, the phantoms were rotated 90° around the z-axis. Subsequently, the test scans were repeated for the rotated phantoms in the third scenario, again using the same three tube current settings. Finally, the retest scans were performed for the rotated phantoms in the fourth scenario. In total, 12 scans were acquired for each phantom (Figure 1).

### 2.3. Scan Reconstructions

A PER with a slice thickness and increment of 1 mm was generated using a Br40 kernel. Additionally, 16 VMER were generated at energy levels ranging from 40 to 190 keV in 10 keV increments using spectral post-processing (SPP) data, also with a slice thickness and increment of 1 mm and a Qr40 kernel. The axial reconstruction images were created, exported, and stored in the Digital Imaging and Communications in Medicine (DICOM) format. These DICOM files were subsequently converted to Neuroimaging Informatics Technology Initiative (NIfTI) format for further analysis.

### 2.4. Segmentation and Radiomic Features Extraction

The NIfTI files were processed using MITK Workbench 2021.10, a dedicated segmentation tool. A clinical radiologist, A.H., with 5 years of experience, performed semi-automatic segmentation for each phantom. The segmentation was based on the PER scan with a tube current of 100 mAs, and regions of interest (ROIs) were delineated for subsequent radiomic feature extraction. Because all PER and VMER images were reconstructed from the same raw acquisition, they shared an identical image geometry and were intrinsically co-registered on a voxel-by-voxel basis. The reference segmentation defined on the 100 mAs PER image—chosen for its high signal-to-noise ratio and the most reliable depiction of the phantom boundary—was therefore propagated unchanged to all PERs and VMERs of the initial (non-rotated) state without any spatial transformation, resampling, or interpolation. Using a single, identical mask for all reconstructions was a deliberate decision to remove inter-segmentation variability as a confounder, so that the measured feature differences reflect the reconstruction type and energy level rather than differences in ROI geometry; this also motivated the exclusion of shape features, which are invariant under a fixed mask. As whole organic phantoms were segmented, boundary and partial-volume voxels represented only a small fraction of the comparatively large ROIs and being identical across all reconstructions, contributed as a constant systematic component to every comparison rather than as a differential bias between energy levels. For the rotated state, the initial segmentation could not be reused, as the phantoms no longer occupied the same voxels. Rather than rigidly transforming the mask, a new and independent reference segmentation was created on the rotated 100 mAs PER image and propagated unchanged to all rotated PERs and VMERs, mirroring the procedure used for the initial state. Re-segmentation rather than mask transformation was chosen because it reflects clinical practice, in which lesions are re-delineated at each examination. Feature extraction was performed with PyRadiomics 3.0.1, whose feature definitions are in substantial accordance with the Image Biomarker Standardisation Initiative (IBSI) [18]. For each phantom, a total of 105 original features were extracted, including shape-based, First-Order Statistics (FO), Gray-Level Co-Occurrence Matrix (GLCM), Gray-Level Size Zone Matrix (GLSZM), Gray-Level Run Length Matrix (GLRLM), Neighboring Gray Tone Difference Matrix (NGTDM), and Gray-Level Dependence Matrix (GLDM) features [19]. The extraction was performed with voxel normalization, resampling to a voxel size of 2 mm × 2 mm × 2 mm, and rebinning with a fixed bin width of 25 Hounsfield units [20]. The Chebyshev distance was set to 1. Isotropic resampling of 2 mm × 2 mm × 2 mm was applied so that texture features, which depend on directional voxel neighborhoods, are computed on equal spacing in all directions, as recommended by the IBSI. A fixed bin width of 25 HU was used rather than a fixed bin count because CT intensities are expressed on an absolute, calibrated scale (HU), for which fixed-bin-width discretization preserves the relationship to the underlying attenuation and improves cross-image reproducibility. Both settings were kept identical to the preprocessing of the source dataset [17] and were applied uniformly to all PERs and VMERs in the same state, ensuring that resampling and discretization contribute as a constant rather than as a differential bias between energy levels.

### 2.5. Statistical Analysis Design

According to a review by Traverso et al. [21] variation in tube current was shown to have no significant effect on feature reproducibility. Therefore, in the present study, the datasets used for each comparison case were created by pooling the values of the extracted radiomic features from the three scans acquired with different tube currents within each scenario. For example, to assess agreement between radiomic features extracted from polyenergetic reconstructions in the test and retest scenarios, the values of each radiomic feature were pooled from the scans acquired at tube currents of 10, 50, and 150 mA in each corresponding scenario. This yielded a total of 48 values for each radiomic feature across the 16 phantoms in each comparison case.

Prior to analysis, normality of radiomic features was assessed using the Shapiro–Wilk [22] test at a 5% significance level. Given the heterogeneous ranges and units of radiomic features, relative metrics were used to evaluate agreement and reliability. Agreement was assessed using the concordance correlation coefficient (CCC) and reliability was evaluated using the intraclass correlation coefficient (ICC) according to McGraw and Wong [23], as described in the Supplementary Materials. A threshold of 0.9 was used to indicate high agreement and reliability. For each comparison case, the number of features exceeding this threshold was reported. CCC confidence intervals are calculated using z-transformation at the 95% confidence level, and ICC confidence intervals are determined using the quantiles of the F-distribution, also at the 95% level. The analysis consists of the following three main parts.

#### 2.5.1. Repeatability Analysis

Repeatability, as defined in the Supplementary Materials, refers to the consistency between independent measurements using the same method on identical subjects under identical conditions. It is assessed by comparing radiomic features of the same reconstructions to evaluate agreement and reliability across test and retest scans.

#### 2.5.2. Reproducibility Analysis

Reproducibility, as defined in the Supplementary Materials, refers to the consistency between independent measurements using the same method on identical subjects but under different conditions or by different raters. It is assessed by comparing radiomic features of different reconstructions. Reproducibility is evaluated in two ways: intra-scan reproducibility, which compares measurements within a test scan to assess intrinsic consistency, and inter-scan reproducibility, which compares test and retest scans to evaluate consistency across different scans.

#### 2.5.3. Agreement and Reliability Analysis of the Average Values of Radiomic Features in Test and Retest Scans, Before and After the Rotation of Phantoms

This section examines the effect of phantom rotation on radiomic features. The average value of all radiomic features for the same reconstructions is calculated for both test and retest scans in the initial and rotated states. Agreement and reliability of these averages are then evaluated.

#### 2.5.4. Selection of Proper ICC Version

For selecting the appropriate version of the ICC, readers may refer to the original papers by Shrout and Fleiss [24] and by McGraw and Wong [23], which define the various ICC forms. In addition, the guidelines by Koo and Li [25] provide a straightforward framework for selecting the appropriate ICC model. The primary objective of this study is to evaluate the reliability of measurements obtained from a fixed set of raters, namely the reconstructions, in terms of absolute agreement. All subjects in the comparison cases are rated by a specific rater from this fixed set. Accordingly, a two-way mixed-effects ICC model is appropriate for all three parts of the study. For both repeatability and reproducibility analyses, no averaging of values is involved. However, in Section 2.5.3 the analysis is based on the average values of two conditions, namely the initial and rotated phantoms.

Taking these considerations into account, the specific ICC model selected for each part of the study, according to McGraw and Wong [23], is as follows:

- Repeatability Analyses: ICC (A, 1).
- Reproducibility Analyses: ICC (A, 1).
- Analyses of Average Values of Radiomic Features in Test and Retest Scans, Before and After Rotation of Phantoms: ICC (A, 2).

#### 2.5.5. Threshold for High Agreement and Reliability

Some references [26,27,28] have suggested specific thresholds for acceptable CCC values depending on the context of their studies. However, the interpretation of correlation coefficients varies across scientific fields, and no universal standard exists for assessing their strength [27]. This variability is particularly relevant for radiomic features, which may differ substantially in scale, units, and numerical range. Accordingly, some other references [29,30] have stated that large positive CCC values indicate good agreement without defining a specific threshold for acceptable or good agreement. In the present study, a CCC value of 0.9 or greater was considered indicative of good agreement. This conservative threshold was selected because values above 0.9 typically reflect a high level of concordance between measurements, with only minimal variation between datasets.

For ICC, several references have proposed a minimum acceptable threshold, while others have defined ranges to classify ICC values as reflecting poor, acceptable, or good reliability [31,32,33]. The threshold for ICC was selected based on the same rationale as that for CCC. Because no universal standard exists for interpreting correlation coefficients, the threshold in this study was determined according to the definition of reliability and ICC, as well as the guideline proposed by Koo and Li [25]. Accordingly, an ICC value of 0.9 or greater was considered indicative of good reliability, reflecting that 90% or more of the observed variance in the radiomic feature values is attributable to true differences between subjects, whereas the remaining 10% or less is due to measurement error.

### 2.6. Technical Infrastructure

The analysis was conducted using R 3.6.3 and Python 3.13.0 within a conda 24.11.3 environment. The Shapiro–Wilk test was performed with the Python package scipy 1.14.1. Lin’s CCC and its confidence intervals (CIs) were calculated using the R package epiR 2.0.19, while the ICC and its CIs were calculated using irrNA 0.2.3.

## 3. Results

In the repeatability and reproducibility studies, shape-based radiomic features showed excellent agreement and reliability. As mentioned earlier, these features were excluded from the analysis.

### 3.1. Normality of Radiomic Feature Values

Before proceeding with the statistical analysis, the normality of radiomic feature values was assessed using the Shapiro–Wilk [22] test. The results indicated that, on average, approximately 73 out of 91 radiomic features (around 80%) do not follow a normal distribution. This non-normality was considered when selecting analysis metrics, which are less sensitive to such deviations. In all scans, the radiomic feature values of VMER at 40 keV exhibited the fewest non-normal features, while VMER at 80 keV showed the most. Detailed results for each scan are provided in the Supplementary Materials.

### 3.2. Repeatability Analysis

The agreement assessment in the repeatability analysis showed that 60 radiomic features of the PER were highly repeatable. Similarly, 61 features were found to be reliable. Overall, about 67% of the radiomic features in the PER demonstrated repeatability (Table 1).

**Figure 2:**
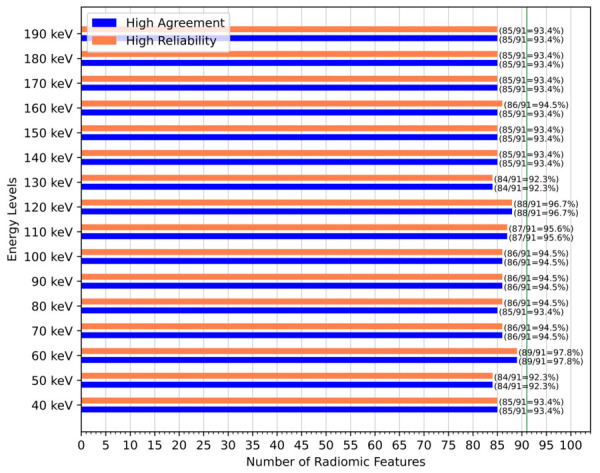
Repeatability of the virtual monoenergetic reconstructions in test and retest scans. Green line shows the total number of analyzed radiomic features.

On average, 85 out of 91 radiomic features (about 93%) from the VMER were found to be repeatable. The 60 keV energy level exhibited the highest repeatability, with 89 out of 91 features (97.8%) demonstrating strong agreement and reliability. In contrast, the energy levels at 50 and 130 keV showed the lowest repeatability, with 84 out of 91 features (92.3%) exhibiting strong agreement and reliability. A total of 83 features (more than 91%) were consistently repeatable across all comparison cases (Figure 2).

### 3.3. Intra-Scan Reproducibility

**Figure 3:**
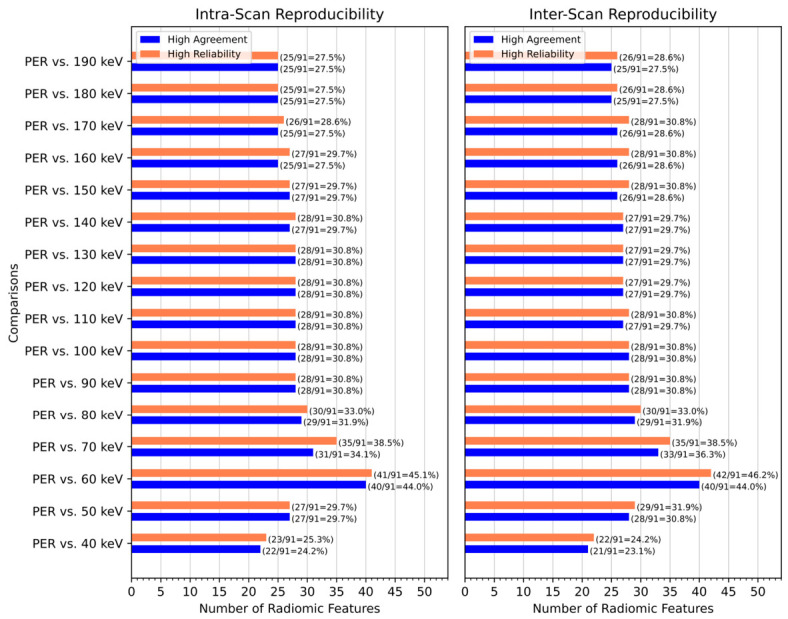
Intra-scan and inter-scan reproducibility of the polyenergetic and virtual monoenergetic reconstructions; PER: polyenergetic reconstruction.

When comparing PER vs. VMER of the test scan for phantoms in the initial state, an average of 27 radiomic features showed high agreement, while 28 features demonstrated high reliability (around 30% of features). The best reproducibility was observed at the 60 keV energy level, with approximately 45% of the features being reproducible. The lowest reproducibility was observed at 40 keV, with only about 25% of the features showing reproducibility (Figure 3).

**Figure 4:**
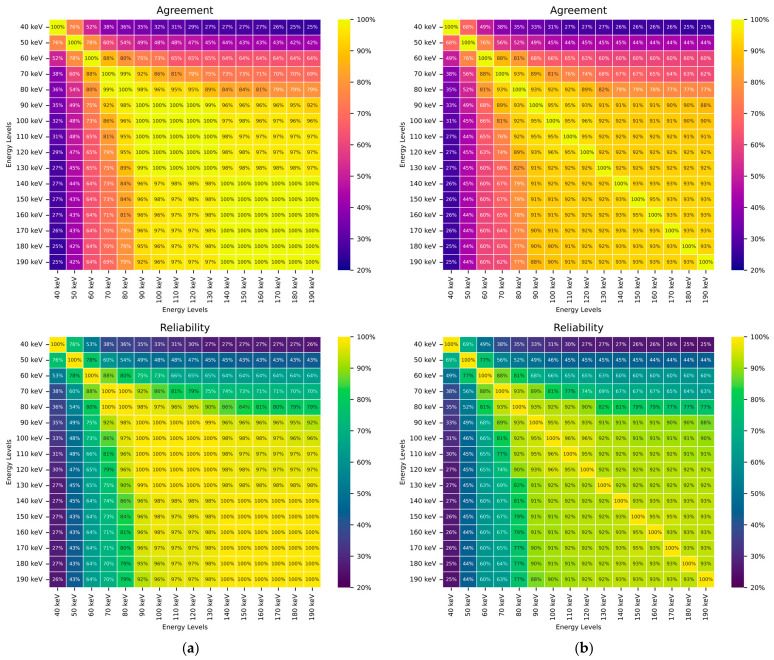
Rounded percentage of reproducible features for each comparison case of virtual monoenergetic reconstructions: (**a**) results for intra-scan reproducibility analysis; (**b**) results for inter-scan reproducibility analysis.

The comparison of different energy levels of VMER showed that, on average, 71 out of 91 radiomic features (around 78%) exhibited high agreement and reliability across 120 comparison cases. Comparisons involving higher energy levels (starting at 90 keV) with minimal differences in energy levels demonstrated perfect reproducibility, with 100% agreement and reliability across all features (Figure 4a). In contrast, comparisons with lower energy levels and larger differences in energy levels showed significantly poorer reproducibility. The lowest reproducibility was observed in comparisons between 40 keV and both 180 and 190 keV energy levels, with only 23 out of 91 (25.3%) features being reproducible.

### 3.4. Inter-Scan Reproducibility

The comparison of PER images from the test scan vs. VMER images from the retest scan for phantoms in the initial state showed that, on average, 27 radiomic features demonstrated high agreement, while 28 features showed high reliability (about 30% of features). Among these comparisons, the 120 kV vs. 60 keV case exhibited the highest reproducibility, with 40 features showing high agreement and 42 features showing high reliability (around 45%). In contrast, the 120 kV vs. 40 keV comparison showed the lowest reproducibility, with only 21 features showing high agreement and 22 features demonstrating high reliability (roughly 24% of features) (Figure 3).

In the 120 comparison cases between the different energy levels of VMER from the test and retest scans in the initial state, an average of 67 radiomic features (approximately 74%) showed high agreement and reliability. The comparison between 100 keV and 120 keV exhibited the highest reproducibility, with 87 out of 91 features (over 95%) being reproducible (Figure 4b). In contrast, comparisons between 40 keV and both 180 and 190 keV showed the lowest reproducibility, with only 23 reproducible features (about 25% of features).

### 3.5. Agreement and Reliability of the Average Values of Radiomic Features in Test and Retest Scans, Before and After the Rotation of Phantoms

The analysis of the average values of radiomic features from the PER, before and after the rotation of the phantoms, revealed that 59 radiomic features (approximately 65%) demonstrated high agreement, and 66 features (around 72%) showed high reliability (Table 2).

**Figure 5:**
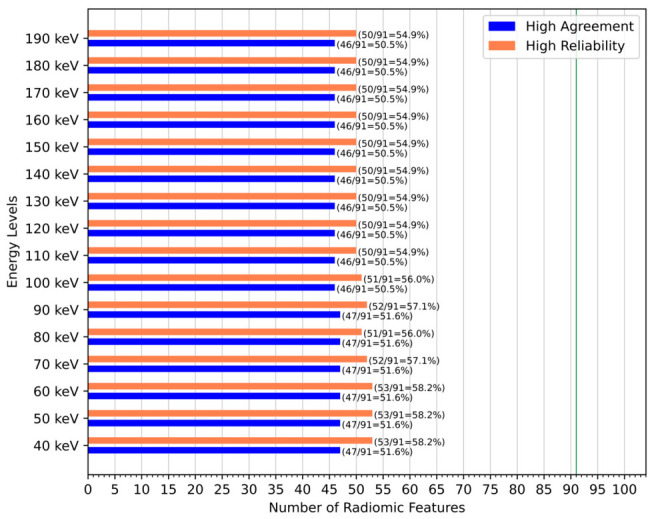
Analysis results of the average values of radiomic features before and after rotation of phantoms for virtual monoenergetic reconstructions. Green line shows the total number of analyzed radiomic features.

The analysis of different energy levels of VMER showed an average of 46 features (roughly 51%) with high agreement and 50 features (nearly 55%) with high reliability. Slightly higher-than-average agreement and reliability from average were observed at energy levels between 40 and 60 keV, with 47 features (about 52%) showing high agreement and 53 features (around 58%) demonstrating high reliability. Conversely, energy levels from 110 to 190 keV exhibited fewer number of features with high agreement and reliability compared to the average, with 46 features (roughly 51%) showing high agreement and 50 features (almost 55%) demonstrating high reliability (Figure 5).

Table 3 summarizes the results of all parts of the statistical analysis, namely repeatability, reproducibility, and the analysis of average values in the radiomics analysis, by presenting the average number and proportion of features showing high agreement and reliability. Table 4 summarizes the most and least stable radiomic feature classes by comparing the proportion of features with high agreement and reliability relative to the total number of features in each class. Detailed results of these analyses, including tables for each comparison case, heatmaps for each radiomic feature class, and the names of the stable features, are provided in the Supplementary Materials.

## 4. Discussion

In this study, the repeatability and reproducibility of images from PER and different energy levels of VMER were evaluated. The results show that radiomic features from VMERs at different energy levels exhibit higher repeatability, with an average of 85 features demonstrating repeatability, compared to only 61 features out of 91 for PER.

VMER-derived radiomic features show similar reproducibility to PER, with about 30% being reproducible in both intra- and inter-scan comparisons. The 60 keV energy level exhibited the highest reproducibility. Intra-scan evaluation showed 71 out of 91 reproducible features, dropping to 67 in inter-scan. Higher energy levels (90 keV and above) or closely spaced levels had better reproducibility. VMER features were generally more reproducible than PER, with intra-scan consistency higher than inter-scan. Phantom rotation had less impact on PER stability than on VMER, with greater stability at lower energy levels (40–60 keV).

These findings were obtained in organic phantoms, which provide a controlled, reproducible model but differ from patients in important respects; they are geometrically static, largely homogeneous, free of iodinated contrast and pathology, and do not reproduce the large or variable body habitus, motion, and tissue heterogeneity encountered in vivo. The stability reported here should therefore be regarded as an upper bound, with somewhat lower agreement and reliability expected in patient data.

Several observations nonetheless have plausible clinical correlates. The higher repeatability of VMER over PER and the better reproducibility at higher (≥90 keV) and closely spaced energy levels are consistent with the lower noise of these reconstructions, indicating that energy-level selection is a relevant determinant of radiomic stability. Comparable trends have already been reported in vivo; Wolf et al. [34] observed greater feature stability at higher and proximate keV levels in human myocardium, and Tharmaseelan et al. [35] found VMER-derived features more stable than non-spectral reconstructions in patient abdominal scans. This concordance suggests the energy-dependent pattern is likely to carry over, at least qualitatively, to clinical datasets.

The superior repeatability of radiomic features also suggests that VMER may mitigate patient-size-dependent beam-hardening and photon-starvation effects that often degrade clinical radiomics. Notably, 60 keV showed the highest reproducibility in this study (71 intra-scan and 67 inter-scan stable features), indicating a favorable operating window in which contrast and texture stability can be jointly preserved [36]. Although virtual monoenergetic images around 70 keV are often considered closest to conventional polyenergetic CT, the difference between 60 and 70 keV is small, and the 70 keV convention is largely a rule of thumb derived from contrast-enhanced biological tissue. Given that our organic phantoms had different material composition and no iodinated contrast, a slightly lower optimum at 60 keV is plausible and remains consistent with expected measurement uncertainty.

The reduction in reproducibility from intra- to inter-scan comparisons likely reflects real-world acquisition variability, such as tube-related fluctuations [37]. At the same time, the high stability maintained at higher energies (≥90 keV) provides a practical alternative for radiomic follow-up in larger patients, where quantum mottle becomes more prominent. However, the finding that phantom rotation affected VMER stability more than PER—especially at 40–60 keV—highlights an important translational challenge: in patients, asymmetric geometric changes across longitudinal scans due to respiration and variable positioning [38] may further affect feature consistency. Therefore, rigorous positioning protocols and/or rotation-invariant radiomic features will be important to minimize positional confounding in longitudinal tumor texture analysis.

A possible additional explanation for the markedly higher repeatability of VMER is the combined effect of reconstruction kernel selection and noise behavior. In this study, VMER was reconstructed with the Qr40 kernel, which is optimized for quantitative spectral analysis, whereas PER was reconstructed with the Br40 kernel. In addition, virtual monoenergetic reconstruction maintains a largely constant noise power spectrum across energy levels, which stabilizes texture features across repeated acquisitions and contributes to the higher repeatability observed for VMER.

Although the identical mask ensures that the same voxels are evaluated in every reconstruction, the object-to-background contrast and image noise vary with the selected energy level, so that the intensity values of partial-volume voxels at the phantom boundary differ across VMER. This energy-dependent boundary behavior is inherent to virtual monoenergetic reconstruction and may contribute to the lower reproducibility observed for low and widely separated energy levels.

In line with this study and its findings, Wolf et al. [34] analyzed the repeatability and reproducibility of radiomic features from organic phantoms and human myocardium using PCCT. They included 12 organic phantoms consisting of four oranges, four onions, and four apples, and 23 patients with coronary computed tomography angiography (CCTA) on PCCT. The organic phantoms were scanned five times in a row, and the image acquisition parameters were identical to the CCTA. For all scans, VMER at six keV levels (40, 55, 70, 90, 120, and 190 keV) were conducted, and segmentations of the whole phantoms and the left ventricular myocardium were extracted. They reported a trend that a higher percentage of radiomic features was stable at higher VMIs (≥90 keV) and VMIs in close proximity of each other. This resonates with the outcomes of our study, as evidenced by the diagrams of reproducibility analyses presented in the Supplementary Materials. They also report an almost identical trend when analyzing the left ventricular myocardium of a patients’ cohort. However, having VMIs close in value did not guarantee better correlation results in this case.

Tharmaseelan et al. [35] evaluated in the early stages of PCCT the radiomics feature stability in abdominal monoenergetic reconstructions on abdominal portal-venous phase scans of 10 patients. They included VMERs of 40 to 120 keV in 10 keV steps, PERs, virtual non-contrast reconstructions, and iodine maps. For spectral reconstructions, 1 and 2 cm 2D and 3D segmentations of the liver, lung, spleen, psoas muscle, air, and subcutaneous fat were obtained, and the radiomics features were extracted. Radiomic features of VMER were more stable compared to non-VMER. The highest average ICC of 0.63 was shown for 3D 2 cm VMER. However, feature stability varied between different organs, ROI sizes, and monoenergetic reconstructions. These results correspond to our findings, which demonstrate that the reproducibility of radiomic features across different energy levels of VMER is greater than the reproducibility observed when comparing VMER with PER.

A review by Traverso et al. [21] indicated that in CT studies, first-order and shape features are generally more repeatable. Among these studies, first-order features were identified as the most reproducible when compared to other texture-specific features. They also noted that among first-order features, entropy was one of the most stable. This finding supports the results of our study, where a significant number of first-order features demonstrated high agreement and reliability, as illustrated in the Supplementary Materials. Notably, six first-order features—90th percentile, energy, mean, median, root mean squared, and total energy—remained stable across all analyses. When we lowered the threshold for agreement and reliability to 0.75, we found that entropy also maintained stability in all analyses. This discrepancy may be attributed to the different statistical approaches and thresholds employed to assess the stability of radiomic, as reviewed by Traverso et al.

The study has several limitations. First, the use of 16 organic phantoms may not fully represent human tissue diversity, limiting the generalizability to clinical settings. The small sample size and variability among phantoms might affect reliability and inflate results. Additionally, the study used a single tube voltage (120 kV) and three tube currents, restricting the evaluation of other common clinical settings. VMERs were only performed across 16 energy levels (40–190 keV), potentially missing broader variability in PCCT and with a different kernel than polyenergetic reconstructions. Moreover, the study is hampered by its single-platform approach, without an external validation set, and through the limited number of phantom types.

Another limitation is that PERs and VMERs were reconstructed with different kernels (Br40 and Qr40, respectively). As the reconstruction kernel directly affects image texture and noise, this difference likely contributes to the limited reproducibility observed between PERs and VMERs, and the two reconstruction types should therefore not be regarded as interchangeable for radiomic analysis without kernel-specific harmonization.

A further limitation is that CCC and ICC were applied by treating reconstruction settings as analogous raters. Although this provides a practical framework for assessing feature consistency across reconstructions, the reconstructions are deterministic image-processing outputs rather than independent observers. Therefore, these metrics should be interpreted as measures of radiomic stability across reconstruction settings, rather than as true inter-rater agreement.

Statistically, the data deviates from normality, which could impact results, despite robust metrics. The use of relative metrics could be refined with absolute metrics for more precise evaluation, and the arbitrary threshold for agreement may distort consistency assessments. Finally, the lack of external validation limits the applicability of the findings to real-world clinical scenarios.

## 5. Conclusions

This study demonstrated that radiomic feature stability in photon-counting CT depends strongly on both reconstruction type and energy level. VMER-derived features showed high repeatability within the same energy level, with substantially more features meeting the criteria for high agreement and high reliability than PER. However, reproducibility between PER and VMER was limited, with only about 30% of features showing high agreement and high reliability, indicating that these reconstruction types are not readily interchangeable for radiomic analysis. In contrast, reproducibility among VMER images reconstructed at 16 different energy levels was, on average, approximately 47% higher than that observed for PER, reaching about 78.5% overall. More specifically, reproducibility of VMER-derived features was highest between neighboring energy levels and decreased as the energy difference increased, particularly for widely separated pairs such as 40 keV versus 180 or 190 keV (see Figure 4). By comparison, PER showed greater stability than VMER in the analysis of average values before and after phantom rotation.

Overall, these findings suggest that radiomic features can be highly stable under identical imaging conditions, but their interchangeability is limited when either the energy level or the reconstruction type changes. Therefore, standardization of reconstruction settings and energy levels is essential when extracting radiomic features from PCCT-derived images.

## Figures and Tables

**Table 1:** Repeatability of the polyenergetic reconstruction in test and retest scans.

Comparison Case	High Agreement		High Reliability	
	No. of Features	Ratio	No. of Features	Ratio
120 kV	60/91	65.93%	61/91	67.03%

**Table 2:** Agreement and reliability of average values of radiomic features extracted from polyenergetic reconstruction in test and retest scans, before and after rotation of phantoms.

Comparison Case	High Agreement		High Reliability	
	No. of Features	Ratio	No. of Features	Ratio
120 kV	59/91	64.84%	66/91	72.53%

**Table 3:** Summary of results of the statistical analysis—the number and ratio of features with high agreement and reliability. PER: polyenergetic reconstruction, VMER: virtual monoenergetic reconstruction, Cases: number of comparison cases, *: average value over all comparison cases.

Analysis		Cases	Agreement		Reliability	
			No. of Features	Ratio	No. of Features	Ratio
Repeatability	PER	1	60/91	65.9%	61/91	67.0%
	VMER	16	85/91 *	94.1% *	85/91 *	94.2% *
Intra-Scan Reproducibility	PER vs. VMER	16	27/91 *	30.4% *	28/91 *	31.2% *
	VMER vs. VMER	120	71/91 *	78.2% *	71/91 *	78.5% *
Inter-Scan Reproducibility	PER vs. VMER	16	27/91 *	30.4% *	28/91 *	31.5% *
	VMER vs. VMER	120	67/91 *	74.1% *	67/91 *	74.3% *
Average Values Before and After Rotation of Phantoms	PER	1	59/91	64.8%	66/91	72.5%
	VMER	16	46/91 *	51.0% *	50/91 *	56.0% *

**Table 4:** Radiomic feature classes representing the most stable and least stable portions of features relative to the total number of features in each class. Cases: number of comparison cases, PER: polyenergetic reconstruction, VMER: virtual monoenergetic reconstruction, FO: First-Order Statistics, GLCM: Gray-Level Co-Occurrence Matrix, GLSZM: Gray-Level Size Zone Matrix, GLRLM: Gray-Level Run Length Matrix, NGTDM: Neighboring Gray Tone Difference Matrix, GLDM: Gray-Level Dependence Matrix.

Analysis		Cases	Agreement		Reliability	
			Highest Stability	Lowest Stability	Highest Stability	Lowest Stability
Repeatability	PER	1	FO	GLSZM	FO	GLSZM
	VMER	16	FO, GLCM, NGTDM	GLDM	FO, GLCM, NGTDM	GLDM
Intra-Scan Reproducibility	PER vs. VMER	16	FO	GLDM	FO	GLDM
	VMER vs. VMER	120	FO	GLDM	FO	GLDM
Inter-Scan Reproducibility	PER vs. VMER	16	FO	GLDM	FO	GLDM
	VMER vs. VMER	120	FO	GLDM	FO	GLDM
Average Values Before and After Rotation of Phantoms	PER	1	FO	GLSZM	FO	GLSZM
	VMER	16	GLRLM	GLSZM	GLRLM	GLSZM

## Data Availability

The dataset forming the foundation of the analysis in this manuscript—the extracted radiomic features of organic phantoms using photon-counting CT—has already been published on the Mendeley Data platform and is available at the following URL: https://data.mendeley.com/datasets/w8pd5995cd/1 (accessed on 29 April 2026) (DOI: 10.17632/w8pd5995cd.1).

## Supplementary Materials

The following supporting information can be downloaded at: https://www.mdpi.com/article/10.3390/tomography12070102/s1, Refs. [21,22,23,24,25,26,27,28,29,30,31,32,33,39]. More information please visit: https://zenodo.org/records/21126504, accessed on 2 July 2026.

## Abbreviations

The following abbreviations are used in this manuscript:

CCTA	Coronary Computed Tomography Angiography
CI	Confidence Interval
CT	Computed Tomography
EICT	Energy-Integrating Computed Tomography
IBSI	Image Biomarker Standardization Initiative
ICC	Intraclass Correlation Coefficient
keV	Kilo-Electron-Volt
kV	Kilovolt
mAs	Milliampere Seconds
No	Number
PCCT	Photon-Counting Computed Tomography
PER	Polyenergetic Reconstruction
ROI	Region of Interest
VMER	Virtual Monoenergetic Reconstruction
VMI	Virtual Monoenergetic Image
