# Supplementary material for: Impact of Different Energy Levels of Virtual Monoenergetic Reconstructions on Radiomic Features Stability in Organic Phantom Imaging Using Photon-Counting CT
Source: Tomography. 2026 Jul 6;12(7):102. doi: 10.3390/tomography12070102 (PMC13417476; doi:10.3390/tomography12070102)
Supplement: Supplementary file 1 [file tomography-12-00102-s001.zip › tomography-4380278-supplementary.pdf]

# Impact of Different Energy Levels of Virtual Monoenergetic Reconstructions on Radiomic Features Stability in Organic Phantom Imaging Using Photon-Counting CT

## Supplementary Material

# Table of Contents

Abbreviations ..... 2
Detailed Statistical Analysis Design ..... 3
Repeatability Analysis ..... 3
Reproducibility Analysis ..... 3
Intra-Scan Reproducibility ..... 3
Inter-Scan Reproducibility ..... 4
Agreement and Reliability Analysis of the Average Values of the Radiomic Features in Test and Retest Scans, Before and After the Rotation of Phantoms ..... 4
Proper Metric Selection ..... 4
Metric for Assessment of Agreement ..... 4
Selection of Proper Reliability Metric ..... 5
Selection of Proper ICC Version ..... 5
References ..... 7

# Abbreviations

| | |
|---|---|
| CCC | Concordance Correlation Coefficient |
| ICC | Intraclass Correlation Coefficient |
| PER | Polyenergetic Reconstruction |
| VMER | Virtual Monoenergetic Reconstruction |

# Detailed Statistical Analysis Design

In the context of statistical analysis, each reconstruction, characterized by a distinct potential or energy level, is treated as a measurement from an individual rater. The algorithm that generates the reconstruction serves as a separate rater, evaluating the scan data by producing images. According to a review by Traverso et al. [1], different tube currents were shown to have no significant effect on feature reproducibility. Consequently, in this study, the analysis incorporates data from all scans, with each phantom providing values from all three tube currents. Specifically, for the 16 phantoms, this results in three values per phantom, leading to a total of 48 values for each analysis case. Based on this premise, the statistical analysis can be designed accordingly.

The statistical analysis can be broken down into three main parts:

1. Repeatability Analysis
2. Reproducibility Analysis
3. Agreement and Reliability Analysis of the Average Values of the Radiomic Features in Test and Retest Scans, Before and After the Rotation of Phantoms

The analysis will report the number of radiomic features that show agreement exceeding the predefined threshold of 0.9 for high agreement in each comparison case. The same approach will be applied to report the number of reliable radiomic features in each comparison case.

Before starting the data analysis, it is important to assess the normality of the radiomic feature values, as some metrics are sensitive to the normality of datasets. Therefore, the normality of the radiomic feature values is evaluated using the Shapiro-Wilk statistical test [2]. For the sake of simplicity, in this study, the repeatability and reproducibility were evaluated only for phantoms in the initial position.

## Repeatability Analysis

In this section, repeatability is assessed by comparing the radiomic features of reconstructions with the same potential or energy level, in line with the definition of repeatability. The objective is to evaluate the agreement and reliability of the extracted features across test and retest scans. For Polyenergetic Reconstruction (PER), repeatability will be assessed in a single case, as only one PER is available. For Virtual Monoenergetic Reconstructions (VMER), repeatability will be evaluated across 16 different energy levels.

## Reproducibility Analysis

In this section, reproducibility is assessed by comparing the radiomic features of reconstructions with different potential and energy levels, in accordance with the definition of reproducibility. Comparisons can be made within a single scan, referred to as *intra-scan reproducibility*, to assess the intrinsic reproducibility of the radiomic features. Alternatively, comparisons can be made between test and retest scans, referred to as *inter-scan reproducibility*. The objective is to evaluate the agreement and reliability of the extracted features for each type of comparison.

### Intra-Scan Reproducibility

Intra-scan reproducibility focuses on the intrinsic reproducibility of the radiomic features in the **test scan**, where the phantoms are in their **initial state**. There are 16 comparison cases for assessing the reproducibility of both PER and VMER, as only one PER and 16 VMER with distinct energy levels are available. To assess the reproducibility across the different energy levels of the VMER, 120 comparison cases are to be evaluated.

## Inter-Scan Reproducibility

Inter-scan reproducibility focuses on the reproducibility of the radiomic features between the **test** and **retest** scans. For assessing the reproducibility of the PER and VMER, there are 16 comparison cases, as there is only one PER and 16 VMER with distinct energy levels. For VMER, reproducibility will be evaluated across 120 comparison cases.

## Agreement and Reliability Analysis of the Average Values of the Radiomic Features in Test and Retest Scans, Before and After the Rotation of Phantoms

This section focuses on studying the effect of phantom rotation on the values of the radiomic features. Since rotating the phantoms does not alter the subjects being measured or the measurement conditions, the impact of this change in orientation does not strictly fit within the predefined statistical definitions of repeatability and reproducibility. However, following the definition of repeatability, this analysis is more closely related to a repeatability study.

To assess the impact of phantom rotation, the average value of all radiomic features for reconstructions with the same potential or energy level is first calculated for both test and retest scans, in both the initial and rotated states. Then, the agreement and reliability of these averages are evaluated.

For PER, only one comparison case is considered, as there is a single PER available. For VMER, 16 comparison cases are included, corresponding to the 16 different energy levels.

## Proper Metric Selection

It is important to highlight that the statistical analysis comprises 306 comparison cases, each involving the assessment of 91 radiomic features. As previously mentioned, shape features are excluded from this analysis because they are dependent on segmentation rather than texture. Given the large number of features and comparisons, applying absolute metrics of agreement (e.g. Total Deviation Index, and Limits of Agreement) and reliability (e.g. Standard Error of Measurement) would not be appropriate for this study.

The primary challenge with absolute metrics is that the thresholds for high agreement or reliability would be heavily dependent on the specific values, units, and ranges of each radiomic feature across various reconstructions and scans. This variability would require separate evaluations for each feature in every comparison and scan, which would go beyond the scope of the study and its intended focus.

Instead, relative metrics provide a more suitable approach for analysing large datasets like this one. Relative metrics focus on the proportional differences between the values, offering a consistent framework for evaluating agreement and reliability regardless of the specific units or ranges of the features. In this study, relative metrics are employed to assess the agreement and reliability of the radiomic features, ensuring a more efficient and consistent analysis across the entire dataset.

## Metric for Assessment of Agreement

In this study, the Concordance Correlation Coefficient (CCC) is used as a relative metric, which is generally robust against moderate deviations from normality [3], to evaluate the agreement between two sets of radiomic feature values.

Some references [4-6] have suggested specific thresholds for acceptable CCC values depending on the context of their studies. However, it is important to note that the

interpretation of correlation coefficients varies across different scientific fields, and there is no universal standard for assessing their strength [5]. This variability is particularly relevant for radiomic features, which can differ widely in terms of values, units, and ranges. Consequently, some other references [3, 7] simply state that large positive CCC values indicate good agreement without specifying a particular threshold for acceptable or good agreement.

In this study, a CCC value of 0.9 or greater is considered to demonstrate good agreement. This threshold is chosen because a CCC value above 0.9 typically indicates a high level of concordance between measurements, meaning that the observed variation between the datasets is minimal.

## Selection of Proper Reliability Metric

In this study, the Intraclass Correlation Coefficient (ICC) is used as a relative metric, which is relatively robust to mild deviations from normality [8], to assess the reliability between two sets of radiomic feature values.

Several references have proposed a minimum acceptable threshold for ICC, while others define ranges to categorize ICC values as reflecting poor, acceptable, or good reliability [9-11]. The selection of the threshold for ICC follows the same rationale as for CCC, as discussed earlier. Given that there is no universal standard for interpreting correlation coefficients, the threshold in this study is determined based on the definition of reliability and ICC, and the guideline established by Koo and Li [12]. Accordingly, an ICC value of 0.9 or greater is considered to indicate good reliability, reflecting that 90% or more of the observed variance in the radiomic feature values is due to true differences between subjects, while the remaining 10% or less is due to errors in the measurement process.

## Selection of Proper ICC Version

The selection of the appropriate ICC version is primarily determined by addressing the following four questions, using either the ICC versions defined by Shrout and Fleiss [13] or by McGraw and Wong [14]:

1. Is there the same set of raters (or measurement instruments) for all subjects?
2. Are the raters randomly selected from a broader population, or is a particular set of raters used in the study?
3. Is the reliability of a single rater being measured, or the mean value of several raters?
4. Is absolute agreement between measurements or consistency among them being sought?

Additionally, the guideline provided by Koo and Li [12] offers straightforward assistance in selecting the correct version of ICC.

It is important to emphasize that the primary objective of this study is to evaluate the reliability of measurements made by a fixed set of raters (reconstructions) in terms of absolute agreement. As described before, all subjects in the comparison cases are rated by a specific rater from this fixed set. Therefore, it is clear that, in all three parts of the study, a version of ICC using a two-way mixed-effects model should be applied. For both the repeatability and reproducibility analyses, no averaged values are involved. However, in the Agreement and Reliability Analysis of the Average Values of the Radiomic Features in Test and Retest Scans, Before and After the Rotation of Phantoms section**,** the analysis is based on the average values of two conditions (initial and rotated).

Taking these details into account, the specific selected ICC model for each part of the study, according to McGraw and Wong [14], is as follows:

- Repeatability Analyses: ICC(A, 1)
- Reproducibility Analyses: ICC(A, 1)
- Analyses of Average Values of Radiomic Features in Test and Retest Scans, Before and After Rotation of Phantoms: ICC(A, 2)

# References


1. Traverso, A., et al., *Repeatability and Reproducibility of Radiomic Features: A Systematic Review*. International Journal of Radiation Oncology*Biology*Physics, 2018. **102**(4): p. 1143-1158.
2. Shapiro, S.S. and M.B. Wilk, *An analysis of variance test for normality (complete samples)†*. Biometrika, 1965. **52**(3-4): p. 591-611.
3. Lawrence Lin, A.S.H., Wenting Wu, *Statistical Tools for Measuring Agreement*. 2012: Springer New York, NY. XVI, 161.
4. McBride, G.B., *A Proposal for Strength-of-Agreement Criteria for Lin's Concordance Correlation Coefficient*. 2005.
5. Akoglu, H., *User's guide to correlation coefficients*. Turk J Emerg Med, 2018. **18**(3): p. 91-93.
6. Mukaka, M.M., *Statistics corner: A guide to appropriate use of correlation coefficient in medical research*. Malawi Med J, 2012. **24**(3): p. 69-71.
7. Choudhary, P.K. and H.N. Nagaraja, *Measuring agreement: models, methods, and applications*. 2017: John Wiley & Sons.
8. Mehta, S., et al., *Performance of intraclass correlation coefficient (ICC) as a reliability index under various distributions in scale reliability studies*. Stat Med, 2018. **37**(18): p. 2734-2752.
9. Atkinson, G. and A.M. Nevill, *Statistical Methods For Assessing Measurement Error (Reliability) in Variables Relevant to Sports Medicine*. Sports Medicine, 1998. **26**(4): p. 217-238.
10. Shrout, P.E., *Measurement reliability and agreement in psychiatry*. Statistical Methods in Medical Research, 1998. **7**(3): p. 301-317.
11. Burdock, E.I., J.L. Fleiss, and A.S. Hardesty, *A New View of Inter-Observer Agreement*. Personnel Psychology, 1963. **16**(4): p. 373-384.
12. Koo, T.K. and M.Y. Li, *A Guideline of Selecting and Reporting Intraclass Correlation Coefficients for Reliability Research*. J Chiropr Med, 2016. **15**(2): p. 155-63.
13. Shrout, P.E. and J.L. Fleiss, *Intraclass correlations: Uses in assessing rater reliability*. Psychological Bulletin, 1979. **86**(2): p. 420-428.
14. McGraw, K.O. and S.P. Wong, *Forming inferences about some intraclass correlation coefficients*. Psychological Methods, 1996. **1**(1): p. 30-46.
